# Bioactive Compounds from Marine Sediment Derived Fungi

**DOI:** 10.3390/md20040242

**Published:** 2022-03-30

**Authors:** Ekaterina A. Yurchenko

**Affiliations:** G.B. Elyakov Pacific Institute of Bioorganic Chemistry, Far Eastern Branch of Russian Academy of Sciences, Pr. 100-letya Vladivostoku, 159, 690022 Vladivostok, Russia; eyurch@piboc.dvo.ru

Marine sediment derived fungi are a very interesting source of biologically active compounds. The development of methods for studying microorganism inhabited sediments has led to the modern view that the biomass of marine sediments, including deep-sea sediments, is very significant [1]. The fungi (eukaryotic microorganisms) of marine sedimental microbial communities are under pressure from bacteria and archaea (prokaryotes) that are superior in biomass and biodiversity [2], and this situation forces them to produce both antibacterial metabolites and substances with cytoprotective properties. Most marine sediment-derived secondary metabolites are polyketides; the second most common are alkaloids and terpenoids combined. This feature is due to the fact that dihydroxynaphthalene melanin pathway plays a special role in the metabolism of fungi for the production of melanin [3], which is of particular importance for the adaptation of fungi to increased levels of UV irradiation and salinity [4,5]. In addition, peptides and meroterpenoids are isolated from marine sediment derived fungi, and it is likely that their importance as biologically active agents will increase with continued research. 

In this Special Issue, Yurchenko et al. reviewed research articles published between January 2016 and November 2020 and considered metabolites from marine sediments and their biological activities. In total, 246 compounds isolated from various locations were analyzed as antiviral, antibacterial, cytotoxic, and cytoprotective agents [6].

The isolation and identification of new metabolites from marine sediment-derived fungi are reported in five articles. Zhang et al. isolated new talaromanloid A, talaromydene, 10-hydroxy-8-demethyltalaromydine and 11-hydroxy-8-demethyltalaromydine, talaromylectone, and ditalaromylectones A and B from *Talaromyces mangshanicus* BTBU20211089 from a sediment sample collected from the South China Sea. Assumptions about the biosynthetic pathways that led to the formation of these compounds as well as seven earlier reported metabolites were also made by the authors. [7]. Yan et al. isolated and identified new metabolites, including a pair of inseparable mixtures of secofumitremorgins A and B, 29-hydroxyfumiquinazoline C, 10*R*-15-methylpseurotin A, 1,4,23-trihydroxy-hopane-22,30-diol, and sphingofungin I, together with six known compounds from the deep-sea sediment-derived fungus *Aspergillus fumigatus* SD-406 collected in the East China Sea. The authors found that the isolated compounds have antimicrobial activity against several human-, aquatic-, and plant-pathogenic microorganisms [8]. Zhong et al. isolated four pairs of new salicylaldehyde derivative enantiomers, euroticins F–I, from *Eurotium* sp. SCSIO F452 collected in the South China Sea. The putative biosynthetic pathway of these compounds as well as their biological activities were also discussed [9]. Matsuo et al. isolated new nitrogen-containing metabolites, hatsusamide A and B, from a culture broth of *Penicillium steckii* FKJ-0213 collected in the Philippine Sea. Moreover, authors found hatsusamide A as a moderate antimalyarian agent [10]. Liu et al. isolated novel diterpenoids longidiacids A and B, two new polyketides, new cytochalasin analogues longichalasins A and B from fungus *Diaporthe longicolla* FS429 collected in the Indian Ocean. Longidiacid A and longichalasin B were discovered as inhibitors of *Mycobacterium tuberculosis* protein tyrosine phosphatase B enzymatic activity [11].

The biological activity of compounds isolated from marine sediment derived fungi are discussed in three research articles. Girich et al. tested *Aspergillus flocculosus*, *A. terreus*, and *Penicillium* sp. from the South China Sea as producers of bioactive compounds and a number of polyketides, bis-indole quinones and terpenoids were isolated. Some of them, especially scytalone derivatives, demonstrated significant neuroprotective and anti-ROS activity [12]. Yan et al. found asperteretone B, aspulvinone H, and (+)-3′,3′-di-(dimethylallyl)-butyrolactone II from *Aspergillus terreus* obtained from the South China Sea as significant glutamic oxaloacetate transaminase 1 enzyme inhibitors in vitro. Aspulvinone H has shown in vitro cytotoxic activity against pancreatic ductal adenocarcinoma cells and in vivo antitumor effect in an SW1990-cell-induced xenograft model [13]. Chingizova et al. reported dual anti-Staphylococcal and anti-inflammatory activities for known cerebroside flavuside B from *Penicillium islandicum* (Aniva Bay, the Sea of Okhotsk). This dual effect allowed flavuside B to protect keratinocytes HaCaT from the toxic effects of *Staphylococcus aureus* in a skin infection model [14].

The materials published in the Special Issue confirm the potential of marine fungi as producers of secondary metabolites with significant pharmacological prospects.
